# Prevalence and causes of mortality in a cohort of patients with type 1 diabetes (T1D) followed at State University of Rio de Janeiro

**DOI:** 10.1186/1758-5996-7-S1-A23

**Published:** 2015-11-11

**Authors:** Ana Paula Almeida Rainho, Marilia de Brito Gomes, Eliete Leao S Clemente

**Affiliations:** 1 Universidade do Estado do Rio de Janeiro, Rio de Janeiro, Brazil

## Background

Type 1 diabetes (T1D) is a chronic disease with an incidence that varies widely throughout the world. Many recent studies have shown an increase in its incidence in developed and developing countries, including Brazil. T1D has become a major health problem and financial burden because of its chronic complications which Results in high direct and indirect costs. The indirect costs are related to early retirement and high mortality at an early age. The objective of our study was to determine the prevalence and causes of mortality in cohort of T1D followed at university outpatient clinic.

## Materials and methods

Our study is a dynamic cohort of patients with T1D started in 1991. The time of follow-up was from 1991 to 2015. Data from patient's vital status were recorded by mail or phone call from December 2014 to June 2015. The cause of death was determined by death certificate and outpatient clinic record and it was stratified as follows: acute complications, chronic microvascular complications, chronic macrovascular complications, other and not defined.

## Results

Among the total of 302 T1D patients (56% females, 61.3% Caucasians), aged 35.4 ±11.9, with duration of diabetes of 20.9±8.8 and age at diagnosis of 15.1±9.2, 244 (80.6%) were confirmed to be alive, 18 (6.0%) had died and 40 (13.2%) the status vital remained unknown. Considering the whole population no difference was observed among patients who had died (33.5±11.0 y), who were alive (35.2±12.2 y) and patients whose vital status were unkown (37.8±10.7 y) p=0.34. Considering the patients who had died, the duration of diabetes until death was 17.9±7.2 yrs., without difference with patients who were alive (p=0.73). The causes of mortality were as follow: acute complications 5 (27.8%), chronic microvascular 7(38.9%), chronic macrovascular 3 (16.7%), not determined 2 (11.1%) and other 1 (5.6%). All deaths from microvascular were from end-stage renal disease, and the deaths from macrovascular were from myocardial infarct. The majority of deaths from acute complications were from diabetic ketoacidosis 3 (60%).

## Conclusion

Our study showed that T1D patients had died at an early age and the causes of mortality were mainly from diabetes-related chronic complications.

